# ‘It wasn’t the strategies on their own’: Exploring caregivers’ experiences of accessing services in the development of interventions for autistic people with intellectual disability

**DOI:** 10.1177/13623613231196084

**Published:** 2023-09-15

**Authors:** Jessica Hughes, Ruth Roberts, Joanne Tarver, Cheryl Warters-Louth, Betty Zhang, Emma Southward, Rachel Shaw, Georgina Edwards, Jane Waite, Effie Pearson

**Affiliations:** 1 Aston University, UK; 2 Expert By Experience, Aston University, UK

**Keywords:** access to services, anxiety, autism, challenging behaviour, intellectual disability, intervention

## Abstract

Autistic individuals with intellectual disability are at greater risk of experiencing anxiety than their non-autistic peers without intellectual disability. Anxiety in this group may present as behaviour that challenges, often leading families to reach out to healthcare or support services. However, many families experience difficulties accessing services and, due to the lack of research into evidence-based anxiety interventions for people with intellectual disability, may not receive individualised support once in a service. This study explored caregivers’ experiences of accessing services for autistic individuals with intellectual disability, and their considerations when developing new interventions for this population. Interviews and focus groups were completed with 16 caregivers of autistic people with intellectual disability. Reflexive thematic analysis was used to develop five themes about service access experiences, and three themes about caregiver considerations for anxiety interventions. Caregivers reported that their experiences of accessing services did not meet their expectations, and considerations for future anxiety interventions were often reflective of this. Interventions being flexible to family circumstances to aid accessibility, the embedding of peer support in services, and skills that can be generalised across the lifespan could be applied when aiming to improve outcomes and develop interventions for this under-served population.

**Lay Abstract:**

Many autistic individuals with intellectual disability experience anxiety, and for those who use few or no words, anxiety may present as behaviour that challenges, such as self-injury and avoiding anxiety-provoking situations. Families report difficulty accessing support from services for autistic individuals experiencing anxiety. Moreover, once receiving support, effective interventions for autistic people with intellectual disability are limited. We completed individual and group discussions with 16 caregivers of autistic people with intellectual disability, to (a) explore their experiences of accessing services for anxiety and/or behaviour that challenges for their child; and (b) understand what matters to caregivers when developing interventions that have been designed for them and the autistic individual with intellectual disability that they support. Caregivers reported that services, in their experience, did not deliver the support that they expected, and that they often needed to ‘fight’ for support. Caregivers considered services and families working together, the inclusion of peer support, and families being offered interventions that are flexible to individual circumstances to be important. These considerations are valuable for clinicians and researchers developing interventions and aiming to improve outcomes for autistic people with intellectual disability and their families.

## Introduction

Autistic individuals with intellectual disability (ID) who speak few or no words may find it difficult to verbally articulate distress; thus, mental health difficulties, alongside distress related to other causes (e.g. pain and discomfort), can present as behaviours such as self-injury, physical ‘aggression’ and avoidance (Edwards et al., 2023; Fitzpatrick et al., 2022). These behaviours can be ‘challenging’ for families and carers to make sense of and pose difficulties in maintaining the safety of the person and those around them, leading families to seek support from services such as parental counselling, child mental health support and respite care (Weiss & Lunsky, 2010). However, caregivers of autistic individuals with ID report difficulties accessing services and interventions for mental health and/or behaviours that challenge (James, 2013; Sapiets et al., 2023). Given the increased prevalence of anxiety (Helverschou & Martinsen, 2011), and greater need to access psychiatric services (Maltais et al., 2020), reported difficulties accessing such services are concerning. Therefore, exploration of experiences of accessing services supporting autistic individuals with severe to profound ID and subsequent areas for improvement is warranted.

Previous research has highlighted disparities between the extent of available service provision, and the reported support required for autistic individuals. Experiences appear to be similar over several years (Galpin et al., 2018; James, 2013; McCarthy et al., 2022; National Autistic Society, n.d.; Sapiets et al., 2023; Sheehan et al., 2018) despite the numerous policies calling for change within this period (NHS England, 2015, 2019). James (2013) identified a need for good communication between professionals and caregivers, and increased consistency of services; limitations of good care were often exacerbated by uncoordinated and bureaucratic service systems. More recently, Sapiets et al. (2023) identified themes including ‘unhelpful professionals’ and ‘complex service systems’ as barriers to accessing early intervention support for families of children with developmental disabilities; facilitators to support included supportive and competent professionals and empowered caregivers. Mental health services were rated the most difficult to access, and such feedback suggests that engaging families in discussions about other services they access for anxiety and/or behaviour that challenges may offer insight into additional facilitators of positive support experiences.

Difficulties accessing services are likely exacerbated by the lack of acceptable and evidence-based psychological interventions for autistic and ID populations (Tschida et al., 2021; Vereenooghe et al., 2018); most mental health research includes autistic people without ID or does not characterise level of ID within the sample (Russell et al., 2019). Recent UK policies have highlighted the need for increased availability of community-based support to reduce long-term residential placements and inpatient admissions (NHS England, 2015). However, there is little research demonstrating what caregivers of autistic individuals with ID view to be important when considering community interventions for anxiety and/or behaviour that challenges. This is concerning, considering many interventions for the ID population are parent-led (McIntyre, 2013), and parental acceptability is linked to improved intervention adherence and outcomes in parent/carer-mediated interventions (Rosenbrock et al., 2021; Rothschild et al., 2022). Understanding what caregivers consider to be important to include in interventions for autistic individuals with severe to profound ID would provide specific insight into the needs of these individuals and their families.

This study uses qualitative methodology to explore caregiver perspectives, obtained through focus groups and interviews, to address the following research questions:

1. What are caregiver experiences of accessing support for anxiety/behaviour that challenges for autistic people with severe to profound ID?
2. What guidance do caregivers provide on intervention features and components, in the context of discussing a proposed caregiver-mediated intervention?

## Methods

Focus groups and interviews with caregivers were carried out to inform, and obtain feedback on, the development of an intervention to reduce anxiety and anxiety-related avoidance behaviour in autistic people with a severe to profound ID (Waite et al., 2022). Caregivers’ experiences of accessing health services for support for anxiety and/or behaviours that challenge for their autistic child were also explored. Details of the specific intervention and pilot testing will be presented elsewhere. The study received ethical approval from Aston University Research Ethics Committee.

### Participants

Participants were caregivers from the United Kingdom and Ireland, recruited from an existing database at Aston University and from an advertisement to the Autistica Discover Network. Caregivers were eligible to take part if they supported an autistic person with severe to profound ID who spoke few or no words.

Seventeen caregivers consented to participate; however, one participant’s data was withdrawn upon request, resulting in 16 participants. Participants were 12 mothers and 4 fathers, and ages ranged from 32 to 64 years (*M* = 46.8 years). Ethical constraints regarding consent meant limited data could be collected for participants’ children over the age of 16. Therefore, ages of the participants’ children were grouped into over/under 16 years, with 14 autistic individuals under 16 years and 2 over 16 years. Participant demographics are provided in Table 1. Race/ethnicity data was not recorded. Fifteen children had a confirmed autism diagnosis and one scored above cut-off for autism spectrum disorder on the Social Communication Questionnaire (Rutter et al., 2003). The Vineland Adaptive Behaviour Scales–Third Edition (VABS-3; Sparrow et al., 2016) was collected for 11 out of 14 autistic people under 16. All individuals scored in the low category of adaptive ability. Where VABS-3 data could not be collected to confirm expressive language levels alongside level of ID, level of ID was confirmed to be within the severe to profound range based on the level of support the individual required in daily life and the individual’s communication ability, as identified through discussions between the researchers and caregiver during recruitment. Eligible families answered ‘no’ to the question ‘is the person you care for verbal? (i.e. more than 30 signs/words in their vocabulary)’.

**Table 1.** Demographic data for parent/caregiver (*n* = 16)^a^ and their child (*n* = 16).

Child age, years	
Under 16, *n* (%)	14 (87.5)
Over 16, *n* (%)	2 (12.5)
Child gender	
Male, *n* (%)	13 (81.3)
Female, *n* (%)	3 (18.7)
Autism diagnosis, *n* (%)	15 (93.8)
Other diagnoses	
Fragile X Syndrome, *n* (%)	1 (6.3)
9q34 deletion, *n* (%)	2 (12.5)
Down syndrome, *n* (%)	2 (12.5)
Caregiver mean age (*SD*), range	46.8 (7.8), 32–64
Caregiver gender	
Male, *n* (%)	4 (25)
Female, *n* (%)	12 (75)
Relationship to child	
Mother, *n* (%)	12 (75)
Father, *n* (%)	4 (25)
Household income	
Less than £15,000, *n* (%)	1 (6.7)
£15,001 to £25,000, *n* (%)	1 (6.7)
£25,001 to £35,000, *n* (%)	3 (20)
£35,001 to £45,000, *n* (%)	2 (13.3)
£45,001 to £55,000, *n* (%)	2 (13.3)
£55,001 to £65,000, *n* (%)	1 (6.7)
£65,000 or more, *n* (%)	5 (13.3)
Highest level of caregiver education	
No formal qualifications, *n* (%)	0 (0)
Fewer than five GCSEs or O-levels, *n* (%)	1 (6.7)
Five or more GCSEs or O-levels, *n* (%)	3 (20)
Three or more A levels, *n* (%)	0 (0)
University degree, *n* (%)	7 (46.7)
Master’s or Doctoral degree, *n* (%)	4 (26.7)

a One caregiver only provided partial responses, which are presented where available.

### Procedure

Participants were given the option to take part in either a focus group of up to four caregivers or a one-to-one interview with a researcher. Nine participants took part in a focus group and seven participants had one-to-one interviews. Both interviews and focus groups followed the same schedule, offering the opportunity for a similar amount of depth, in accordance with other focus group research (e.g. Pellicano et al., 2014). Focus groups and interviews will now be collectively referred to as interviews. The interviews were completed using video conferencing software, an accessible and feasible way of conducting remote qualitative research (Eigege et al., 2022).

The interviews followed a semi-structured format; the first section concerned families’ experiences of accessing services (voluntary and statutory) on behalf of their child for anxiety and/or behaviour that challenges. Caregivers were asked about the types of services they had accessed, the nature of any support received, what they had found helpful, less helpful and the reasons for the support finishing. If caregivers had not accessed any services for anxiety and/or behaviour that challenges, the reasons for this were explored. The second section sought feedback from families about a partially developed caregiver-led intervention aiming to reduce anxiety in autistic children with severe to profound ID. Caregivers were given information on the background, evidence-base and core psychological components underpinning the intervention, and the planned structure and delivery. Families were asked if they thought the intervention components were appropriate, what they liked and disliked, if there was anything they would change and any barriers they envisioned.

### Data analysis

All interviews were anonymised, transcribed verbatim, and analysed using the step-by-step approach of reflexive thematic analysis outlined by Braun and Clarke (2006). The following analysis and dissemination involved caregivers, to ensure that interpretations made maintained ecological validity (Sweeney et al., 2012), and the voices of caregivers remained the most salient aspect of the research.

Authors J.H. and R.R. familiarised themselves with the transcripts prior to analysis. J.H. open-coded transcripts, identifying data relevant to the broad research questions, while remaining open to the generation of unrelated and unexpected codes. A ‘codebook’ was created, with each code defined through descriptions and data from the transcripts. The codebook was used to collaboratively recode the transcripts by two authors (J.H. and R.R.), allowing for new interpretations and discrepancies to be identified and discussed openly. Following the initial coding stages, a caregiver Patient and Public Involvement (PPI) group (C.W.L., B.Z. and E.S.) discussed the initial codes with researchers. Discrepancies between codes were discussed with support from direct quotes from the transcripts to enable the development of a consensus. The PPI group received a 90-min overview of thematic analysis and the process of coding and theme generation prior to discussing the data.

After refinement of code names and descriptions, codes were placed into groups with shared meaning by authors (J.H., R.R. and E.P.). The groups of codes were shared with the PPI group in a second session where the formulation of overarching themes, subthemes, and identification of more salient themes occurred. The group formulated broad descriptors of the themes and discussed the relationships between themes to develop a narrative. Relevant key quotes were identified to further aid theme descriptions.

### Community involvement

As previously highlighted, three caregivers of autistic children with ID were involved in the analysis and interpretation of data presented in this article. The caregiver PPI group contributed to the development of this article, providing feedback on drafts. The caregiver PPI group were recruited through an existing participant database held at Aston University and had not been involved in the study as participants.

## Results

**Figure 1.**
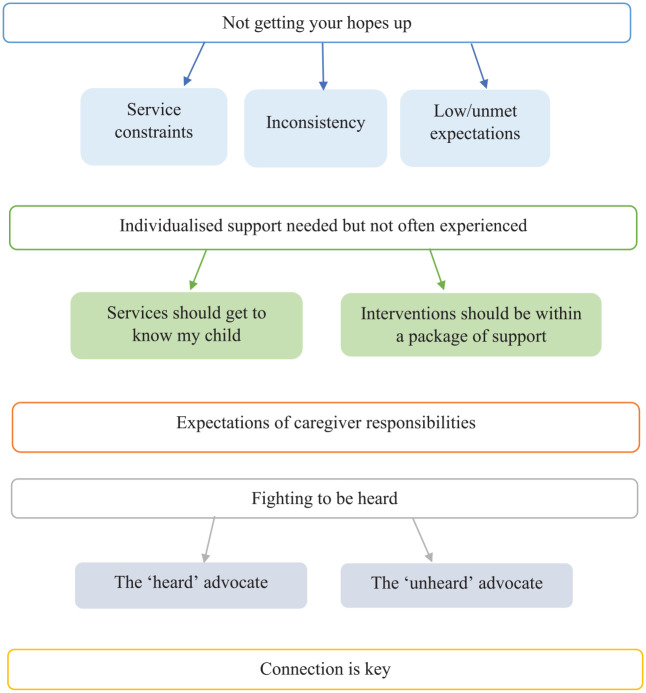
Caregiver experiences of accessing services for anxiety and/or challenging behaviour.

**Figure 2.**
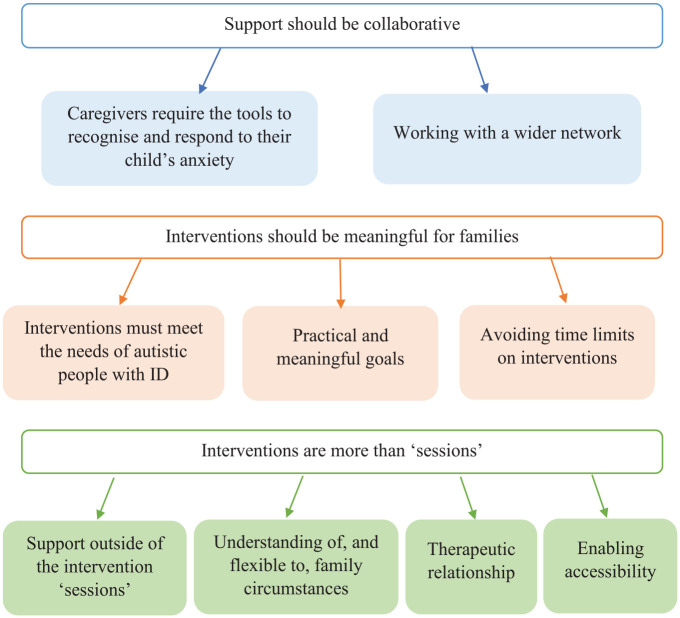
Caregiver considerations for interventions for their autistic child with ID.

In total, eight themes were generated in the analysis, grouped under the two a priori research questions. Five themes with seven subthemes addressed the question of caregivers’ experiences of accessing services (Figure 1). Three themes with nine subthemes addressed the question of caregivers’ guidance for interventions (Figure 2).

### Caregiver experiences of accessing services for anxiety and/or behaviour that challenges

#### Not getting your hopes up

Most families reported significant difficulties obtaining support for their child. The expectations that families had of the type of support they would be offered often contrasted the actual service they received:

it was almost like there’s a diagnosis and instead of, at that point I thought everything would kick into gear, [. . .] but in fact the opposite happened, in that the box had been ticked and then he fell off.

With many families reporting experiences of expectations that were not met, the families who expressed positive experiences often framed these within the context of ‘luck’:

I have been lucky in the sense that X has a really good paediatric consultant, and she’s very supportive and she does listen to what I say, [. . .] she’s always acted upon what I am saying.

It is possible that the sense of ‘luck’ resulted from the generally unmet needs of families accessing services, leading to a perspective that positive experiences were improbable, and expectations should remain low. The inconsistency many families reported appeared to contribute to the improbability of positive experiences, suggesting even once receiving support, staff turnover or service limitations may lead to inconsistent support:

usually in the NHS it is very sort of like infrequent and you know as we all know for our children it’s consistency and continuity, you know, keeping things in a regular pattern that really helps maintain that you know regulate their mood and sort of anxiety.

As highlighted, routine and consistency are perceived as essential for autistic children with ID so inconsistent support creates an additional concern. Inconsistency was present in other areas of service access; a disparity between local service provision was recognised by families, which was a source of frustration:

I see other people who are benefitting in ways that I’m not and I’m struggling with that

The funding constraints of statutory services were viewed as a contributing factor to the unmet needs of families, with many acknowledging how service provisions were stretched thinly:

There’s a very good additional needs nurse but again I don’t really use her that much because obviously she’s limited to what she can offer.

The experiences of caregivers highlighted perceived risks of engaging with a service when unsure of the outcome, consistency and resulting impact this could have on their child’s well-being. Where services may not be available when needed or be able to provide the consistency and depth of knowledge that caregivers perceive as necessary, having low expectations may offer an understandable way of coping with uncertainty.

#### Individualised support needed but not often experienced (expectation vs reality)

The need for individualised support was central to caregivers’ experiences of both difficult and successful encounters with statutory services. Diagnostic overshadowing and false assumptions about diagnoses held by professionals were reported as barriers to good care. Assumptions were sometimes attributed to a lack of understanding, particularly within non-mental health services (e.g. physical health):

They [medical professionals] just see Down syndrome first, can’t understand why the child isn’t bubbly and friendly and then when you explain about the autism and then on top of that she has anxiety, but it just doesn’t seem to compute

Assumptions being made about a child’s behaviour often coincided with the provision of ‘one size fits all’ strategies based on diagnoses, rather than person-centred care informed by assessment. Caregivers felt in-depth assessments were essential for professionals to develop a greater knowledge and understanding of their child, however this was not often experienced:

OT [occupational therapy] give you a sheet, you know, exercises you can do, breathing exercises [. . .] so it’s kind of like, it’s almost like a pack . . . a one thing fits all, I don’t know. They don’t address anxiety or behaviours at all.

Medication prescribed for anxiety was sometimes reported as helpful; typically in the context of a wider package of support (e.g. alongside therapeutic interventions and thorough assessment of need):

it wasn’t the strategies on their own because we’ve got X the right medication we, I think as he got used to his sister, his behaviour got a bit better, anyway, there with the strategies as well, so I think it was one of a number of things that we put in place to kind of make X’s life better.

However, caregivers frequently reported the prescription of medication without clinicians taking the time to understand the individual’s personal circumstances. Caregivers were concerned that medication was at times the only offer, or was suggested before alternative support had been considered:

in the absence of not having any psychologist [. . .] because I think they cut a load of posts or something, he suggested drugs straight away.

Caregivers discussed that when medication was offered in these circumstances, it did not appear to be based on individualised care, but to avoid lengthy waits or other service limitations. Such decisions were met with a sense of frustration:

it took at least 6 months before we even got an appointment with the psychiatrist and at that point I was asked what sort of medication was I thinking she might need, and I was like no we want coping strategies [. . .] and then I think it was an ‘oh okay then I will see you in another month’, and this kind of went so on, and nothing really changed.

#### Expectations of caregiver responsibilities

Caregivers reported a high level of responsibility when supporting their child with anxiety and/or behaviour that challenges. Responsibilities included sourcing comprehensive information about their child’s often complex difficulties/diagnoses, and at times financing support for anxiety and/or behaviour that challenges. These experiences appeared to result from the constraints present within service delivery, and in some cases the absence of available statutory services:

They [services] give you a pamphlet and that’s, what is that is that you know, it’s something about the size of an envelope and you’re expected to just figure it out yourself.

we started a programme at home in the end in the absence of nothing else, and we borrowed money on our house in the years when you could borrow money on your house, and bankrupted ourselves basically trying to help him.

The lengths that families report going to when seeking support for their child are clearly not sustainable as a model of support delivery, with reported methods of obtaining funding not possible for many families.

In addition, services were felt to be challenging to navigate, requiring a high level of expertise. Caregivers felt professionals working in services also found this a challenge, adding to the sense of impossibility around accessing services successfully:

And there’s this assumption which drives me insane that you know exactly how it all works – they don’t know how it works, the people that are in the system don’t know how it works, how the hell you’re expected to know God I don’t know.

Caregivers may willingly carry out their responsibilities to improve their child’s quality of life, but they reported the *expectation* of knowledge and responsibility could not reasonably be applied to all families:

I am a parent that’s vocal, and I think it’s working in the care sector and supporting people, that’s made me like it, but when you talk to some parents that haven’t got that confidence, a lot of them will just sit there and take what somebody says to them and just accept it, and you have to learn to fight for things.

Expecting families to have certain levels of knowledge about services, material resources available to support their child, and the confidence to fight for support was therefore suggested to benefit families who were able to meet those expectations, thereby increasing already-present inequality, and subsequent inequity.

#### Fighting to be heard

Family advocacy was frequently referred to by caregivers attempting to obtain appropriate support for their child, which was often described as a ‘battle’ or a ‘fight’. This process risked taking precedence over the needs of the child they were advocating for:

it’s kind of like you’re lost in the battle of trying to get people to listen, but at the end of the day there is a child sitting there that needs help.

Such advocacy was particularly important for caregivers of autistic children with ID, who may find it challenging to articulate their distress verbally, creating additional barriers to accessing services:

We have an older child, she needed [mental health service] last year, and the only route seemed to be, go to a GP and go sit in a hospital and then get to see a mental health doctor, and that’s the child who is verbal [. . .] you think how am I gonna manage this with a child who can’t – and if they’re in pain, their reaction could be to laugh rather than just go ‘ouch’ you know, kind of different ways to manage all of that.

The value placed on the voices of caregivers appeared to be disproportionately lower than that placed on the voices of professionals. Caregivers highlighted this when discussing their experiences of seeking referrals to services for anxiety and/or behaviour that challenges; their requests were sometimes refused, or ignored until other professionals advocated on their behalf:

We met the X [research] team, and X who did communication, and X did the clinical assessments, so it wasn’t a diagnosis, but an assessment [. . .] so then we brought that report back and they said ‘oh I see’.

For some families, professional advocacy was essential, where the set-up of services did not allow for caregiver advocacy alone:

I’m trying to refer myself over and I’m getting just sent round in circles, one person ‘oh we didn’t receive that we didn’t receive that’, ‘oh it’s got to be with this person you can’t refer yourself you have to have a professional do it’.

This experience is concerning, given that professional advocacy was also suggested to increase barriers to support. Though professional advocacy can be valuable for obtaining support for families, the reliance on this can perpetuate devaluing of caregivers’ voices:

X’s paediatrician [. . .] didn’t believe that she was autistic [. . .] they kind of not blocked but made it very difficult to access services because of what they, their opinion that they put in writing, but when we were assessed by [mental health service], X scored really really highly so it was obvious to everyone else, but not the paediatrician.

#### Connection is key

Connectedness between systems and families was a common theme present where caregiver’s reported positive experiences of accessing services. Having a point-of-contact was reassuring to have, even if not used, and contacts included both professionals, and parent/carer networks:

but it was reassuring to have, you know, a mobile number and email address that we didn’t have to go right back to the start of the system and apply.

With families reporting experiences of not being heard, and of inconsistencies present when accessing and receiving services, it is possible that caregivers were not fully confident that positive experiences would be sustained. A point-of-contact within, or external to systems, therefore may have provided reassurance that families would not need to repeat their stories, and may experience greater consistency, in contrast to the below experience:

I find that the amount of repetition – explaining, explaining X and his behaviours and so on to various different agencies over and over and over again so that you feel like every time you go to agency A you explain it once, or agency B comes to you, you explain it again and there’s this ever continuing sense of get that far, get knocked back [. . .],there’s no central co-ordination.

Systems not being connected appeared to lead to caregivers not feeling heard, and lengthy, inefficient, processes. When multiple systems, such as education and NHS teams, communicated and worked together, support was delivered more efficiently:

it was a co-ordinated approach from all the departments and then would have the meetings at school where they bring everyone together you know the lady from [mental health service], the social worker [. . .] so everyone knew the input that everyone else was given to, you know getting X to a better place, it wasn’t disjointed.

### Caregiver guidance for interventions

#### Support should be collaborative

When caregivers were asked about anxiety interventions for their autistic child, their considerations often reflected what was missing in their own experiences when they had attempted to seek support. Although services were highlighted as assessing and offering support for current difficulties, caregivers emphasised the importance of families obtaining skills that are generalisable to future goals or difficulties:

I find that that you know there would be something that would be a particular issue and you might get some help to kind of you know work on it [. . .] then something else will happen you know a few months down the line so I think the last bit is really important, making sure that parents have got the tools to kind of work through the process themselves and come up with things they might try.

Future-planning was felt to be an important factor currently lacking in existing support that tended to focus on reducing specific behaviours. Obtaining a generalised understanding of the functions of behaviours that may re-occur in different circumstances, would in the long term enable the identification and prevention of distress, and associated behaviour that challenges.

Collaboration was discussed as important for ensuring professionals listened to the views of caregivers, who often have expert knowledge of their child and the needs of the family. However, caregivers were aware that this must not be an expectation for families who may need additional support to make sense of their child’s anxiety:

we’re assuming a little bit in this-our conversation that we know the source of the anxiety, what would be interesting in your study would be actually if it turns out to be something else. Because that might happen as well, because for a non-verbal child or young person, there’s a level of assumption on the part of the parent that they know the reason.

Families raised the importance of wider collaboration when taking part in interventions, through involving wider networks, such as other family members and systems (e.g. education). Caregivers suggested that by incorporating wider systems, the burden on singular family members could be reduced. It was suggested that taking a whole family approach would enable a more naturalistic (and straightforward) application of the intervention:

siblings of family can become part of the positive reinforcement of what they’re doing, or the, you know, showing that this is the best way to go forward, take a deep breath, or explain that they’re part of the story as well as the intervention too, and siblings are the best teachers you find, as well.

#### Interventions should be meaningful for families

Caregivers reported experiencing a ‘one-size-fits-all’ approach to interventions when accessing services. Meaningful interventions specifically for families of autistic children with severe-profound ID were considered essential, due to the complexity of identifying anxiety within this group:

he has anxiety when he cannot communicate how he feels or what he needs, that sometimes comes out in more frustrated behaviours, so we’ve got the sensory dysregulation as a result of anxiety, but sometimes the anxiety comes through dysregulation so it can work both ways, which is complex.

Caregivers referred to interventions that were not considered to be person-centred, leading to goals based on compliance rather than quality of life; however, caregivers also reported the need to find a balance between avoiding anxiety-provoking situations and being sensitive to the needs of autistic people:

there is a balance because there’s times where he doesn’t want to do something and we have to, and I don’t count that as a cruelty it’s just tough kiddo you know we’re going to have to just break through this barrier so there is a, there is a place for that.

Caregivers discussed the practicalities of achieving anxiety-related goals when anxiety can be complex to identify and reported that goals planned within interventions should be feasible, to reduce the likelihood of families feeling disheartened. Caregivers also highlighted how interventions should deliver content that is meaningful, and therefore engaging. Non-prescriptive content, where caregivers had choices and options, was referred to as desirable:

I think that kind of flexibility would be important that you know we were going through some area and me being able to say actually, I really don’t think this applies to you know my child but this other area we started discussing, yes, can we discuss that in lots more detail?

Caregivers discussed the lack of depth obtained from time-limited input and short-term interventions, particularly when behaviours are complex, and present differently to non-autistic individuals without ID. Longer interventions, that could be flexible and extended, if necessary, would offer the opportunity to embed skills more effectively:

I believe like as a parent I would be worried that if we’d gone like one or two steps backwards, if it’s limited to 16 weeks it’s really going to detriment everything else and we’re not going to get to the end of it before the 16 weeks is up.

The meaningfulness of interventions appeared to be associated with the development of skills that could accompany families throughout their child’s life. The well-being of families was suggested to be impacted when a sense of ‘failure’ is experienced during an intervention, and could be mitigated by providing sufficient time, and setting feasible goals.

#### Interventions are more than ‘sessions’

Despite specific intervention strategies being presented (in line with the methodology of the interview delivery), families focused on the wider context in which interventions were delivered, and the values held by services responsible for providing interventions. There was a sense that the context in which ‘session’-based interventions were offered needed to be therapeutic and accessible. Caregivers discussed the wide range of responsibilities and barriers present for families, such as caring for siblings of their autistic child and relying on public transport, and how services needed to respond compassionately and flexibly to families experiencing difficulties committing to interventions:

[child with ID]’s got a younger sister and an older brother who don’t have anxiety issues and aren’t autistic and it’s just you know doing things with them, and holidays, [. . .] I don’t think we’re the only family with that, to keep it continually week in week out might just sometimes for reasons you can’t foresee, just it doesn’t always work that way however high you try and prioritise something.

The caregiver-professional relationship was considered important for providing consistency. Having the opportunity to build relationships enabled open discussions, and the role of the clinician was highlighted as being responsible for helping caregivers make sense of their existing knowledge, as well as offering new insights:

As parents we’re sort of like the experts because, obviously, we know our children really well and so it’s kind of like having someone draw it out for you with the insights and things you have but just might not realise.

Having access to support outside of intervention ‘sessions’, such as resources, parent networks and direct contact with clinicians, was important for families, offering the opportunity for families to share important information in a timely way, access support ‘in the moment’ and apply interventions effectively:

on a phone call as well – things might not come into your head, but you could think oh, I’ll just pop that into an email and – you always think of something after the phone call.

Finally, the opportunity to obtain complex information from interventions was desirable for some caregivers:

it’s, that could be, that, that hat appeals to me, maybe because I’ve got an academic background – but I really like that, I like to learn about X’s condition I like to demystify it, so from my point of view that’s very appealing.

However, though desirable for some families, the ‘academic’ nature of interventions, alongside other commitments, was suggested to risk excluding families who may need greater levels of support to fully access and understand intervention content:

you are gonna have to be fairly switched on as a parent to engage in that kind of programme. Parents may also have other siblings, that means that that they cannot devote that kind of time to it, so I think I think it would, it would attract a certain type of parent.

Due to the time commitment and risk of excluding families with additional responsibilities, it was suggested that the use of digital platforms may provide a way for caregivers to engage in interventions more efficiently alongside everyday commitments:

whether there’s some sort of app-based way of recording things so that so that you have like a series of just four or five tick boxes but you can sort of go yeah that one, that one [. . .], the data that’s been collected would be more, possibly more, effective.

In highlighting the context in which interventions are delivered, caregivers demonstrated that though important, the therapeutic relationship should be more than rapport building and knowledge sharing. Being flexible to using alternative methods of ‘intervention’ delivery was suggested to aid the effectiveness of an intervention for both the family and the service provider.

In summary, families’ experiences of accessing services appeared to differ from their expectations, with many families reporting significant difficulty gaining access to a specialist service for anxiety and/or challenging behaviour. Caregivers raised several considerations that aided, or would aid, successful service access. Caregivers also expressed the impact of systems and networks outside of the family-service provider relationship, and how these contribute to the effective engagement and support for an autistic individual with ID and their family.

## Discussion

This study explored caregivers’ experiences of accessing services for anxiety and/or behaviour that challenges for their autistic child with ID due to anxiety frequently presenting through behaviours that challenge (Edwards et al., 2023), as well as caregivers’ guidance for the development of anxiety interventions. Experiences reported in this study are consistent with previous studies focusing on broader service access (Galpin et al., 2018; James, 2013; Sapiets et al., 2023). Most caregivers reported services had not met their child’s needs; the constant ‘battle’ to obtain appropriate support appeared to lead to a sense of hopelessness around the possibility of having a positive experience. Caregivers emphasised the need for interventions based within systems that understand specific family circumstances and that are willing to explore ways to enable individuals to access interventions meaningfully. Despite methodological and demographic differences, our study aligns with previous qualitative work by Galpin et al. (2018), whose themes included the desire for connection between families and services, flexible support opportunities, and the concerning expectations placed on families. The consistencies across studies are encouraging, demonstrating transferability of findings across wider population groups, one of the quality markers of qualitative research (Lincoln & Guba, 1985).

Caregivers placed responsibility on services for encouraging engagement and access, in contrast to research on service perspectives, where engagement difficulties are often framed within an individual’s choices and perceptions of services (Martin et al., 2005; Starke, 2011). Having a trusting therapeutic relationship with professionals was highlighted as important caregivers in the current study. As therapeutic rapport is important for successful intervention outcomes (e.g. Kazdin & Whitley, 2006), focusing on improving this could encourage re-engagement of families with services, particularly when they have had negative previous experiences accessing services (Weiss & Lunsky, 2010). Prior to delivering interventions, researchers and clinicians may wish to consider whether enough time has been given to allow for the development of a meaningful therapeutic alliance, particularly for caregivers of autistic individuals with severe to profound ID, where caregiver report and engagement is often relied upon (Edwards et al., 2023).

Although this article has focused on caregiver experiences, it is important to acknowledge here that autistic people with ID should also have the opportunity to advocate for themselves. The greater resources and training needed to support this advocacy often leads to a reliance on caregiver report only, with limited involvement of the autistic individual with ID in decision-making (Doherty et al., 2020). When applied to broader service delivery, professionals providing longer appointment slots to interact with, and observe autistic people with ID may reassure families that service providers’ decisions are person-centred, while reducing the reliance on caregiver report. It is encouraging that steps are being taken to highlight the legal requirement of such adjustments in public services (Public Health England, 2020) through the NHS Reasonable Adjustments Digital Flag currently in development (NHS Reasonable Adjustments Digital Flag, n.d.). Despite person-centred care existing in guidelines for a number of years (e.g. STOMP; NHS England, 2016), it is rarely implemented routinely in practice (Sheehan et al., 2018). Policy makers and researchers should regularly review the real-world effectiveness of new guidance to ensure if reflects the experience of those accessing services.

Perceived inequity across families was highlighted; caregivers described expectations placed upon them that they believed could be successfully managed by some families, but not all. Caregivers indicated the presence of unhelpful expectations from systems regarding caregivers’ access to significant financial support, ability to obtain complex information about a child’s difficulties, and knowledge of how to navigate complex systems. In line with this, Saunders et al. (2015) suggested that 51% of families are expected to reduce work hours to support their autistic child with ID, despite 52% experiencing additional financial difficulties. The perceived expectations on families to advocate for their child and personally finance support were voiced as resulting from overstretched services unable, or unwilling, to provide support. With there being several criticisms that policies such as the NHS Long Term Plan (NHS England, 2016) have failed to increase support for community services (Taylor, 2021), it is understandable that there may be a mismatch between the expectations of families, and service capacity. However, families were concerned the expectation placed on caregivers regarding extensive advocacy may serve to further marginalise families. The salience of caregiver advocacy when obtaining service provision is concerning given evidence suggesting that individuals with lower health literacy, most often from marginalised groups, are less likely to access services (Sentell & Braun, 2012; Sudore et al., 2006).

A comprehensive understanding of their child was an ‘expectation’ perceived to be required of families by services. However, many families perceived that they had been offered ‘one-size-fits-all’ interventions aimed towards families of autistic children with or without ID, alongside minimal information following their child’s initial diagnosis. Therefore, caregivers emphasised the need for interventions to acknowledge and build on the existing understanding of families. Caregivers also emphasised a need for specific information about the complexities of supporting their autistic child with ID prior to difficulties with anxiety and/or behaviour that challenges becoming unmanageable for families.

Evidence-based early-intervention programmes for families of autistic children, such as Cygnet (Stuttard et al., 2016) and the EarlyBird Programme (Shields, 2001), are offered across many statutory services for families of newly diagnosed autistic children. However, many families seek a diagnosis following concerns regarding anxiety and/or behaviour that challenges (Crane et al., 2016; Guinchat et al., 2012), and anxiety is not the primary focus of these programmes. Therefore, future research might consider whether manualised interventions that provide caregivers with more in-depth knowledge and skills specifically with regards to anxiety, may be beneficial alongside autism psychoeducation programmes. Targeted approaches focused on anxiety may be important given that caregivers are often relied upon to identify when their child may need additional support and the high prevalence of anxiety in this group (Bakken et al., 2010; Helverschou & Martinsen, 2011). This may enable families to generalise understanding obtained to a broader range of situations in their child’s life once input from services ends. Such programmes are beginning to be offered to professionals, for example the Anxiety Module delivered through the Autism Education Trust (2007).

When caregivers reported positive experiences, a sense of connectedness was apparent; between families and services, different services, and families and other caregiver-led networks. Families reported more efficient and effective service provision when different services communicated. The positive impact of connection between systems for families is not unknown, and recent policies behind the Integrated Care Systems model (Department of Health and Social Care, 2022) champion reducing the need for families to repeat their stories and navigate systems that do not communicate well with each other. The themes identified in this study demonstrate ways that a sense of connectedness can be fostered, offering solutions for services in the absence of government policies being fully implemented (Charles et al., 2018; Sheehan et al., 2018; Taylor, 2021). Connectedness was present when diverse groups of professionals met together to discuss a child’s needs, co-ordinating their support and sharing information with a family/individual. By meeting together consistently and regularly, the burden of advocacy was reduced for families. A service point-of-contact for families was reassuring, providing a sense of services being accessible. Outside of statutory support services, several families reported accessing, or being connected to, a parent-carer network, and families felt that this would be useful to incorporate in future interventions.

Research demonstrates that caregiver ‘pre-treatment’ social relationships facilitate therapeutic relationships and child intervention outcomes (Kazdin & Whitley, 2006). Further research demonstrates the expert knowledge held by families of children with ID, the solidarity in which that knowledge is delivered to other families, and the impact of peer support on building caregiver confidence and coping skills (Batchelor et al., 2020; Kruithof et al., 2020; Shilling et al., 2014). Therefore, embedding parent-carer networks within services and interventions (i.e. caregiver peer-support) could offer an opportunity to benefit the well-being of families, while improving intervention outcomes, in addition to sharing evidence-based knowledge more widely to families who may not be accessing specialist support. Where implemented, it is important that such public involvement is evaluated and disseminated widely, as the impact of public involvement is often poorly recorded in research (Larkin et al., 2015) and therefore difficult to justify with empirical data.

Many of the themes raised are consistent with previous findings; caregivers’ hopes and expectations for support are often reported to be beyond the capacity of stretched and underfunded statutory services. It is possible that feelings of frustration from families, but also professionals struggling to meet expectations (Ee et al., 2022; Stenfert-Kroese et al., 2012), may expand the divide between families and services. Caregivers reported a difference in the value of professionals’ voices, where professionals were able to advocate for families more successfully than the families themselves; though helpful and often well-intentioned, there were also occasions where families perceived that this greater power led to professionals becoming a barrier to accessing services. It is possible that professional advocacy, when given greater influence than caregiver advocacy, may serve to perpetuate existing power inequities between caregivers and services, leading to continued experiences of disempowered families. Implementing change, such as incorporating the views of families in determining areas of support for an autistic individual with ID may lead to resistance from more dominant levels (i.e. professionals) within the system (O’Shea et al., 2019). The differences between caregiver expectations and service capacity, alongside power differentials reported, may help to explain why the experiences of families receiving services do not appear to reflect the changes proposed in ‘co-developed’ services and policy (James, 2013; Sheehan et al., 2018). Such reflections demonstrate how necessary it is for services to formally embed the contribution and voices of individuals with lived experience at every stage of service-development, ensuring their views are reflected in systems from a bottom-up perspective.

### Strengths and limitations

This study was originally conceived as an initial piece of work in a larger intervention study, which then yielded a richer dataset suitable for standalone publication. With this in mind, we have included the voices of caregivers wherever possible, and maintained core INVOLVE (2012) values such as transparency and responsiveness. This has enabled our research to remain centred around the families that services and interventions intend to support. The extent of public involvement could have been broadened in line with policies (e.g. INVOLVE, 2012), which provide detailed information about the values embedded within ‘good’ participatory research.

Skeens et al. (2022) suggests that social media recruitment strategies may lead to the underrepresentation of marginalised groups in research, such as those with lower socio-economic status or from ethnic minority groups. Therefore, the use of social media for recruiting participants (alongside existing participant databases) in this study makes it difficult to ascertain the extent to which the voices of individuals from these groups may have been reflected. In addition, this research may not transfer cross-culturally due to not collecting race/ethnicity data. As a result, the findings from this study should be interpreted with caution. Future research should identify specific cultural considerations for interventions, particularly considering the increasing discourse regarding the application and acceptability of Westernised models within other cultural/ethnic groups (Nielsen et al., 2017; Rodgers-Sirin et al., 2017; Vicary & Bishop, 2005).

Finally, it is possible that the wide age range of the participants and their children may have impacted the guidance that caregivers provided; depending on whether their child was accessing child or adult services, and the developmental age of the child, opportunities for support may have been different. In some instances, families may have been reflecting on services accessed several years prior to taking part in the study, that may differ from current service provision. Future research in this area would benefit from specifying the services accessed by families and timelines of service access when obtaining background information, alongside standardised measures of anxiety and/ behaviour that challenges, to aid with the interpretation of interview data.

## Conclusion

Our study has identified themes within the context of services in the United Kingdom and Ireland specifically for anxiety and/or behaviour that challenges. This research provides a range of ideas that can aid researchers and clinicians considering how to improve the acceptability of new interventions for families of autistic children with severe ID, and highlights areas of difficulty accessing services for anxiety and/or behaviour that challenges. The wider context of stretched service capacities leading to the provision of support that is not person-centred appeared particularly salient when considering future interventions. Utilising the expertise of families, and service providers collaborating with families when setting goals, was considered important for meaningful outcomes. Caregivers highlighted flexibility, accessibility and supportive service delivery as important components of caregiver-led interventions. The incorporation of caregivers as co-researchers in the current analysis has enabled us to maintain the perspective of a key-stakeholder group within our research, dissemination, and intervention development. This article offers a detailed account of how public involvement can be successfully incorporated within research beyond participation in studies, and its methods can be applied within health research to a wide range of populations outside of the field of autism and ID, to build more effective, person-centred services for all.
